# A *Legionella pneumophila* Effector Protein Encoded in a Region of Genomic Plasticity Binds to Dot/Icm-Modified Vacuoles

**DOI:** 10.1371/journal.ppat.1000278

**Published:** 2009-01-23

**Authors:** Shira Ninio, Jean Celli, Craig R. Roy

**Affiliations:** 1 Section of Microbial Pathogenesis, Yale University School of Medicine, Boyer Center for Molecular Medicine, New Haven, Connecticut, United States of America; 2 Laboratory of Intracellular Parasites, Rocky Mountain Laboratories, National Institute of Allergy and Infectious Diseases, National Institutes of Health, Hamilton, Montana, United States of America; Tufts University School of Medicine, United States of America

## Abstract

*Legionella pneumophila* is an opportunistic pathogen that can cause a severe pneumonia called Legionnaires' disease. In the environment, *L. pneumophila* is found in fresh water reservoirs in a large spectrum of environmental conditions, where the bacteria are able to replicate within a variety of protozoan hosts. To survive within eukaryotic cells, *L. pneumophila* require a type IV secretion system, designated Dot/Icm, that delivers bacterial effector proteins into the host cell cytoplasm. In recent years, a number of Dot/Icm substrate proteins have been identified; however, the function of most of these proteins remains unknown, and it is unclear why the bacterium maintains such a large repertoire of effectors to promote its survival. Here we investigate a region of the *L. pneumophila* chromosome that displays a high degree of plasticity among four sequenced *L. pneumophila* strains. Analysis of GC content suggests that several genes encoded in this region were acquired through horizontal gene transfer. Protein translocation studies establish that this region of genomic plasticity encodes for multiple Dot/Icm effectors. Ectopic expression studies in mammalian cells indicate that one of these substrates, a protein called PieA, has unique effector activities. PieA is an effector that can alter lysosome morphology and associates specifically with vacuoles that support *L. pneumophila* replication. It was determined that the association of PieA with vacuoles containing *L. pneumophila* requires modifications to the vacuole mediated by other Dot/Icm effectors. Thus, the localization properties of PieA reveal that the Dot/Icm system has the ability to spatially and temporally control the association of an effector with vacuoles containing *L. pneumophila* through activities mediated by other effector proteins.

## Author Summary

The survival of intracellular pathogens often involves the modification of the host vacuole in which the pathogen resides. This can be achieved through the function of effector proteins that are delivered into the host cell cytoplasm using specialized transport machinery. In the case of *Legionella pneumophila*, the bacterium that causes a severe pneumonia known as Legionnaires' disease, a type IV secretion system, termed Dot/Icm, delivers a number of proteins into host cells, resulting in altered trafficking of the *L. pneumophila*–containing vacuole. The mechanisms by which effector proteins are spatially and temporally regulated in the host cell to facilitate the survival of the pathogen are not well understood. In this work, we report the identification of several *L. pneumophila* effectors encoded in a genomic region of high plasticity, among them the protein PieA. We demonstrate the Dot/Icm dependent recruitment of PieA to the *L. pneumophila* vacuole and show that the protein binds to the cytoplasmic face of the vacuole as a result of *L. pneumophila*–induced modifications to this vacuole. Our findings demonstrate that the association of an effector with host vacuoles can be spatially controlled through activities mediated by other effector proteins.

## Introduction

*L. pneumophila* is the causative agent of a severe pneumonia called Legionnaires' disease [1],[2]. In the environment it can be found in fresh water reservoirs [3], in a very large spectrum of environmental conditions [4]. In these environments *L. pneumophila* resides within protozoan hosts, where it is able to survive and replicate [3]. A large number of protozoa species can provide a habitat for *L. pneumophila*, among them *Acanthamoeba castellanii*, *Hartmanella* sp. and *Naeglaria* sp. [5]. When humans come in contact with aerosolized contaminated water sources, *L. pneumophila* can access human alveolar macrophages. The bacterium is engulfed by these cells, where it is able to proliferate, and may cause severe disease [6]. *L. pneumophila* is not transmitted between individuals [3] and is therefore thought to have evolved to survive within its Protozoan environmental hosts, and only infect humans as an accidental pathogen.

To survive within eukaryotic cells, *L. pneumophila* requires a type IV secretion system designated the Dot/Icm system [7],[8] that delivers bacterial effector proteins into the host cell cytoplasm [9],[10]. The Dot/Icm system is crucial for the ability of the bacterium to remodel the vacuole in which it resides by preventing delivery of the vacuole to lysosomes [11], and promoting recruitment of endoplasmic reticulum (ER)-derived vesicles to this vacuole to create a unique organelle in which the bacterium survives and replicates [12]–[16]. To date, four *L. pneumophila* serogroup1 isolates have been fully sequenced. These are the Philadelphia1 strain [17], which was derived from the original isolate obtained from the eponymous outbreak at an American Legion convention in 1976 [1], the Lens and Paris strains [18], an epidemic and endemic strain, respectively, isolated in France, and the recently completed Corby strain (GeneBank number CP000675). Sequence comparison revealed a high degree of genomic plasticity, with a large number of strain-specific genes found in each genome [18].

Using genomic data in conjugation with genetic and biochemical methods, many Dot/Icm substrate proteins have been identified [18]–[29]. The function of most of these substrates remains unknown, however, for some effectors biochemical and genetic studies demonstrate activities important for the biogenesis of an organelle that is permissive for *L. pneumophila* replication (reviewed in [30]). The number of substrate proteins identified to date is higher than was initially predicted, and it is not yet clear why so many effectors are required for the survival of the bacteria.

Genomic plasticity and effector abundance could be related to the versatile lifestyle of *L. pneumophila*. These bacteria can survive within a variety of protozoan hosts found in different environments. Because natural environments probably support a defined subset of protozoan hosts, it can be predicted that *L. pneumophila* strains that have evolved in different environments would possess slightly different sets of effector proteins that best facilitate the survival within their environmental hosts. As a first step in addressing this hypothesis, we have focused our investigation on a chromosomal region that displays a high degree of plasticity among the sequenced *L. pneumophila* genomes. We show that effectors of the Dot/Icm system are abundant in this region and demonstrate that one of the effectors encoded in this region is recruited to vacuoles containing *L. pneumophila* by a process requiring Dot/Icm-dependent modifications to the vacuole surface.

## Results

### Identification of lpg1965 as a substrate of the Dot/Icm secretion system

In previous work aimed at identifying novel *L. pneumophila* effectors, a screen was conducted using the Dot/Icm component IcmW as bait in a yeast-two-hybrid system. The screen was successful at identifying several effectors [26]. Further analysis of data generated in that screen has led to the identification of an additional protein fragment capable of interacting with the IcmW protein. This fragment consists of amino acids 715 to 988 of the protein encoded by open reading frame (ORF) lpg1965. A calmodulin-dependent adenylate cyclase (Cya) gene fusion approach was used to test whether the lpg1965 gene encodes a Dot/Icm-translocated substrate protein [31]–[33]. Because Cya enzymatic activity is very low in the absence of calmodulin, this enzyme is inactive in bacterial cells, and is activated when delivered into eukaryotic cells. Fusion of Cya with a translocated effector results in delivery of the hybrid protein into host cells, resulting in a dramatic elevation in cAMP levels. Infection of CHO cells with wild-type *L. pneumophila* expressing the Cya-lpg1965 fusion protein resulted in an increase in cAMP that was three logs above the background levels found in uninfected control cells. When a *dotA* mutant devoid of a functional Dot/Icm system was used, cAMP levels were similar to background levels, indicating that translocation of lpg1965 is mediated by the Dot/Icm system. Because lpg1965 was identified as an IcmW-interacting protein, dependency of the IcmS-IcmW complex for efficient translocation of lpg1965 was tested. As demonstrated for other IcmW-interacting substrates of the Dot/Icm system [26], translocation of lpg1965 was highly dependent on the IcmS-IcmW protein complex (Figure S1).

### The lpg1965 gene is found in a chromosomal region of high genomic plasticity

**Figure 1:**
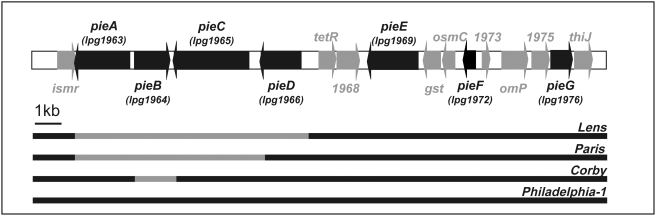
High genomic plasticity in the region encoding lpg1965. The Philadelphia1 genomic region flanking lpg1965 (*pieC*) was analyzed to obtain the specific genomic context of *pieC*. ORFs of novel translocated effectors are shown in black. Below is a low-resolution sequence alignment of this region, to scale, of all four sequenced genomes. Regions absent from the genomes of Lens, Paris and Corby are highlighted with grey bars.

Analysis of other sequenced *L. pneumophila* strains revealed that lpg1965 is absent in the Lens and Paris genomes. Genomic plasticity in the chromosomal region encoding lpg1965 was apparent upon local sequence alignment between the four available genome sequences (Figure 1). We decided to investigate the genomic region delineated by the housekeeping genes encoded by ORFs lpg1962 (peptidyl-prolyl cis-trans isomerase, (*ismr*)) and lpg1977 (ThiJ protease, (*thiJ*)) to determine whether other effector proteins are present. Several genes that reside within this chromosomal region are found in all four strains, where they share extremely high sequence identity, and then there are multiple genes that are absent from one or more of the genomes. One mechanism that could account for genomic plasticity within this region is the acquisition of genetic material by horizontal gene transfer, followed by incorporation of the foreign DNA into the genome [34]. Genetic material incorporated by horizontal gene transfer typically has a different GC content compared with the average GC content of the receiving genome [35]. When compared to the average genomic GC content of 38.3%, lpg1965 and its neighboring genes that are not present in all four strains have a significantly lower GC content of 30.4% (lpg1963), 27.3% (lpg1964), 33.3% (lpg1965) and 33.2% (lpg1966). Although this analysis supports the hypothesis that these genes were acquired through a process of horizontal gene transfer, validation of this hypothesis requires further analysis. Regardless of the mechanism, these data indicate that lpg1965 is located in a region where genomic rearrangements have occurred.

### Multiple proteins translocated by the Dot/Icm system are encoded in the lpg1965 region

**Figure 2:**
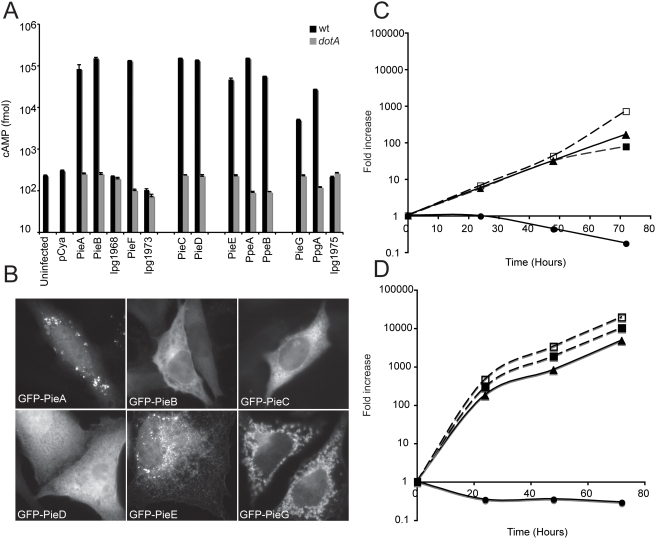
Pie proteins are translocated substrates of the Dot/Icm system and show distinct localization patterns when produced in eukaryotic cells. (A) CHO FcγRII cells were infected, at an MOI of 30, with *L. pneumophila* strains wild-type (black bars) or *dotA* (grey bars) harboring plasmids expressing Cya fusion with the indicated proteins. One hour after infection cells were lysed and cAMP was extracted and quantified as described under Materials and Methods. Levels of cAMP were also determined for uninfected cells (uninfected), or cells expressing Cya alone (pCya). Each bar represents the mean cAMP value obtained from triplicate wells±standard deviation. (B) Epifluorescence micrographs of CHO FcγRII cells expressing the indicated Pie proteins N-terminally fused to GFP, demonstrating unique subcellular distribution phenotypes. (C,D) *L. pneumophila* growth rates were determined in mouse bone marrow-derived macrophages (C), and in the protozoan host *A. castellanii* (D). Intracellular growth of strain SN179 (open squares) was compared to that of strain SN178 (closed squares), wild-type *L. pneumophila* strain Lp01 (triangles), and Δ*dotA* mutant strain CR58 (circles). Each time point represents the fold increase in the mean number of viable bacteria recovered from triplicate wells. The Pie proteins were not essential for the intracellular multiplication of *L. pneumophila* in these cell types.

The observation that the Dot/Icm substrate encoded by lpg1965 was located in a region of genomic plasticity suggested a location where other potential substrates of the Dot/Icm system might reside. To directly test whether additional proteins in the lpg1965 genomic region are Dot/Icm translocated substrates we fused Cya to the amino terminus of nine predicted proteins encoded in this region that were either novel or contained eukaryotic-like domains, and to three proteins encoded elsewhere on the chromosome that were predicted paralogues of proteins encoded in the plasticity region. This analysis revealed ten additional substrates of the Dot/Icm system (Figure 2A). Thus, these genes encode Pie (Plasticity Island of Effectors) proteins that are translocated substrates of the Dot/Icm system. Proteins within the region of genomic plasticity were designated PieA to PieG. Proteins outside of the Pie region were designated PpeA and PpeB, for the two translocated PieE paralogues, and PpgA for the translocated PieG paralogue. Similar to lpg1965 (PieC), translocation of the other Pie proteins was reduced greatly in a mutant strain of *L. pneumophila* deficient in the IcmSW protein complex (Figure S3). Even with the observation that these *pie* genes encode proteins with a functional C-terminal secretion signal recognized by the Dot/Icm system, expression of the *pie* genes was analyzed by reverse-transcription-PCR (RT-PCR) to ensure that these were not pseudogenes. These data show all the *pie* genes are expressed by *L. pneumophila* (Figure S2).

In Table 1 the Pie proteins and paralogues are organized into families based on amino acid identity. The degree of homology between the different family members was calculated using the multiple sequence alignment software ClustalW [36]. Proteins PieC and PieD share 14.7% sequence identity and 22.6% similarity. PieE shares 17.3% and 20.7% identity with PpeA and PpeB respectively, and 29.4% and 33.1% similarity with these proteins, respectively. PieG shares 15.7% and 16% identity with lpg1975 and PpgA respectively, and 23.2% and 25.4% similarity, respectively.

**Table 1:** Pie protein families and their homologues.

Philadelphia-1^1^	Paris^2^	Lens^3^	Corby^4^	Homology Domains^5^
PieA (lpg1963^6^)			lpc1442	Coiled coil
PieB (lpg1964)				
PieC (lpg1965)			lpc1443	Coiled coil
PieD (lpg1966)^7^	lpp1947		lpc1446	Coiled coil
PieE (lpg1969)	lpp1952	lpl1941	lpc1452	Coiled coil
PpeA (lpg1701)	lpp1666	lpl1660	lpc1130	Coiled coil
PpeB (lpg1702)	lpp1667	lpl1661		Coiled coil
PieF (lpg1972)	lpp1955	lpl1950	lpc1459	Coiled coil
lpg1975				
PieG (lpg1976)	lpp1959	lpl1953	lpc1462	RCC1
PpgA (lpg2224)				RCC1

1 GeneBank accession number AE017354.

2 GeneBank accession number CR628336.

3 GeneBank accession number CR628337.

4 GeneBank accession number CP000675.

5 Homology domains were identified using the SMART [57] and CD-Search [58] tools.

6 The original ORF name is indicated in parenthesis.

7 Each family of parologous proteins is placed in a single cell.

### The Pie proteins are not essential for the intracellular multiplication of *L. pneumophila*

*L. pneumophila* strain SN178 is derived from parental strain Lp01 and is deficient in nine of the translocated Pie proteins and related paralogues. SN178 has in-frame chromosomal deletions removing the genes *pieA*, *pieB pieC*, *pieD*, *pieE*, *pieG*, *ppeA*, and *ppeB*. Insertional inactivation of the *ppgA* gene in SN178 resulted in the strain SN179, which is a mutant deficient in ten of the Pie proteins and related paralogues. *A. castellanii* was infected with Lp01, SN178 and SN179, and intracellular growth of these strains was compared to an isogenic Dot/Icm-deficient strain having a mutation in *dotA*. These data indicate that both SN178 and SN179 replicate as well as the parental strain Lp01 in *A. castellanii* (Figure 2D). The fold increase in colony-forming units (cfu) recovered from cells infected with Pie-deficient *L. pneumophila* was similar to the number recovered from cells infected with the parental strain Lp01. As expected, the *dotA* mutant did not replicate in these cells. Similar results were obtained when replication was measured in bone marrow-derived macrophages from an A/J mouse (Figure 2C). Thus, a strain deficient in the repertoire of Pie proteins and related paralogues has no measurable intracellular growth defect in macrophages or protozoan host cells, indicating that these proteins do not play an essential role in establishment and maintenance of a vacuole that supports replication of *L. pneumophila* in cell culture conditions.

### Differential localization of Pie proteins in eukaryotic cells

Several of the Pie proteins contain predicted eukaryotic homology domains (Table 1). Putative coiled coil regions are found in PieA, PieC, PieD, PieF, and in the PieE family. This domain is predominantly found in eukaryotic proteins where it participates in the establishment of protein-protein interactions involved in a wide range of cellular processes including membrane tethering and vesicle transport [37]. Another eukaryotic homology domain identified is the RCC1 motif found in PieG and the related protein PpgA. RCC1 is a guanine nucleotide exchange factor for the Ran-GTPase, which is involved in cell cycle control and other cellular processes [38]. The presence of these putative domains in the Pie proteins suggests that once within the host cells, Pie proteins might function to mimic and manipulate cellular processes to facilitate the intracellular survival of *L. pneumophila*. Because subcellular localization of effectors can provide important insight into their biochemical functions, CHO cells were transfected with plasmids encoding GFP fusions of different Pie proteins to examine the distribution of these protein in mammalian cells. As shown in Figure 2B, GFP-Pie fusion proteins had different subcellular localization properties. There were several Pie proteins that appeared to localize to intracellular membranes. GFP-PieA was concentrated on vesicular structures in the perinuclear region of the cell. GFP-PieE displayed an ER-like reticulate pattern, and GFP-PieG localized to small vesicular-like structures throughout the cell (Figure 2B). None of the Pie proteins disrupted the structure of the Golgi apparatus when overproduced (data not shown), which is a phenotype observed for a number of other Dot/Icm effectors [24],[25],[39]. Thus, Pie proteins have unique subcellular distribution phenotypes that could relate to their ability to target different host proteins and possibly vesicular transport pathways.

### PieA is recruited to the *L. pneumophila* vacuole

**Figure 3:**
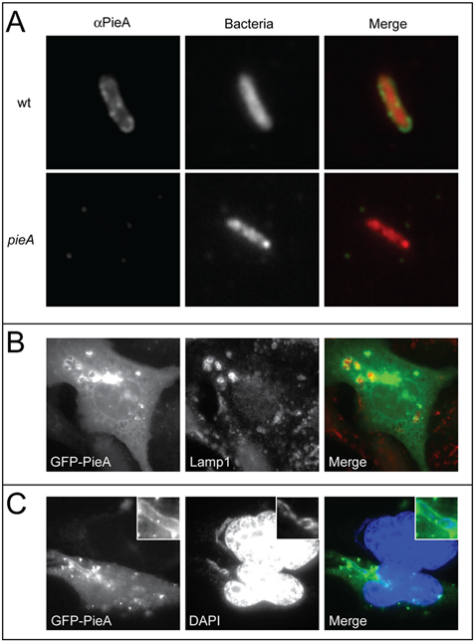
PieA associates with vacuoles containing *L. pneumophila*. (A) Representative epifluorescence micrographs of *L. pneumophila* vacuoles isolated from U937 macrophage-like cells infected with bacteria expressing the fluorescent protein DsRed. Endogenous PieA was detected on vacuoles isolated from cells infected with wild-type *L. pneumophila* using a PieA-specific antibody and FITC-labeled secondary antibodies. No PieA staining was detected on vacuoles from cells infected with a *pieA* mutant strain. CHO FcγRII cells transiently expressing the fusion protein GFP-PieA were (B) fixed and stained for the lysosomal marker LAMP-1, revealing GFP-PieA staining in regions of clustered lysosomes, or (C) infected with wild-type *L. pneumophila* for seven hours, and stained with DAPI to identify bacterial DNA and host cell nuclei. Vacuoles containing *L. pneumophila* are magnified in the inset of each image. GFP-PieA was observed in association with vacuoles containing *L. pneumophila*.

The localization of PieA during infection was investigated further to independently address whether Pie proteins are translocated into host cells during infection. A polyclonal antibody specific for the PieA protein was used to determine whether PieA is found on vacuoles containing *L. pneumophila*. Vacuoles were isolated from U937 macrophage-like cells two hours after infection with *L. pneumophila*. PieA staining was evident on vacuoles containing wild-type bacteria (Figure 3A). No staining was observed on vacuoles containing a *pieA* mutant (Figure 3A). PieA staining was conducted in the absence of permeabilization, and under conditions where the majority of the vacuoles remain intact. Thus, the PieA associated with the vacuoles corresponded to protein on the cytoplasmic face of the vacuole.

**Figure 4:**
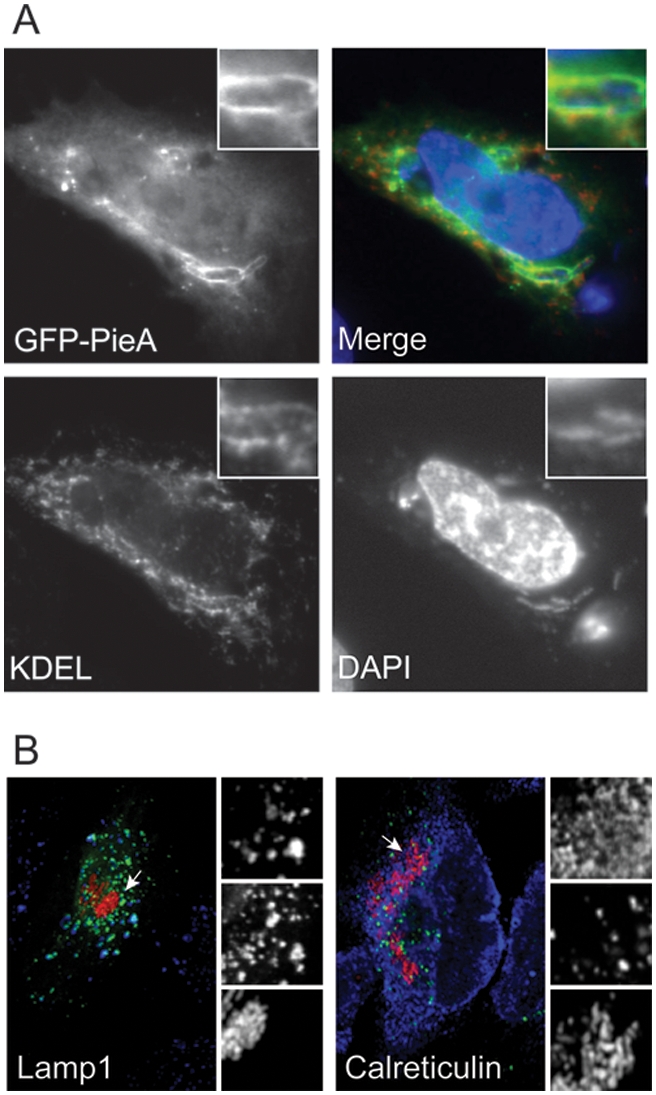
GFP-PieA and KDEL co-localize on the membrane of vacuoles containing *L. pneumophila*, but not on those containing the ER-resident bacterium *B. abortus*. (A) CHO FcγRII cells transiently expressing the fusion protein GFP-PieA were Infected with wild-type *L. pneumophila* for seven hours and stained for the ER marker KDEL to identify the membrane of vacuoles containing *L. pneumophila*. Vacuoles containing bacteria are magnified in the inset of each image. GFP-PieA is found decorating the *L. pneumophila* containing vacuole where it co-localizes with the ER marker KDEL. (B) HeLa cells were transfected to express GFP-PieA and infected with *B. abortus* strain DsRedm-2308 for 24 hours before processing for confocal microscopy analysis. Bacteria (appear red) do not recruit GFP-PieA (appears green) to their replicative vacuoles. GFP-PieA remains associated with LAMP1-positive vesicles (appear blue, left panel) and does not co-localize with the ER marker calreticulin (appears blue, right panel) around the *B. abortus*–containing vacuole.

In cells ectopically producing GFP-PieA there was a clustering of LAMP-1-positive late-endosomal/lysosomal vesicles (Figure 3B). The GFP-PieA protein was found in association with these LAMP-1-positive vesicles. These data suggest that PieA overproduction leads to an alteration in the morphology of host endocytic compartments. Cells producing GFP-PieA were infected with *L. pneumophila* to see if PieA overproduction interfered with any cellular processes important *for L. pneumophila* trafficking and growth. Surprisingly, there was a redistribution of GFP-PieA observed in cells infected with *L. pneumophila*. The GFP-PieA protein was found circumferentially localized to vacuoles containing *L. pneumophila* (Figure 3C). The observed redistribution of the protein upon infection is unique to PieA, and was not observed for any of the other GFP-Pie fusion protein (data not shown). The GFP-PieA staining on vacuoles containing replicating *L. pneumophila* delineated the membrane surrounding the bacteria. Anti-KDEL staining was used to visualize ER proteins with this retention motif, and showed that vacuoles containing *L. pneumophila* that stained positive for GFP-PieA also stained positive with anti-KDEL (Figure 4A). Thus, the GFP-PieA-positive organelles containing *L. pneumophila* have the expected properties of the specialized ER-derived vacuoles that support *L. pneumophila* replication.

GFP-PieA co-localization with ER markers was observed only with the *L. pneumophila*-containing vacuoles in infected cells, suggesting that protein recruitment occurs in response to a pathogen-mediated alteration in the vacuole. To investigate whether pathogen subversion of the ER to create a vacuole that permits replication was sufficient to induce relocalization of PieA to an ER-derived vacuole, GFP-PieA producing cells were infected with *Brucella abortus*, which similarly to *L. pneumophila* requires a type IV secretion system to create an ER-derived vacuole that supports intracellular replication [40]. GFP-PieA showed partial co-localization with LAMP-1-positive compartments in *B. abortus*-infected cells, but no co-localization of GFP-PieA with the ER marker calreticulin was detected in these cells, and no co-localization of GFP-PieA was observed with the *B. abortus*-containing vacuole (Figure 4B). These data suggest that intracellular *L. pneumophila* induce a specific modification to the vacuole in which they reside, and that this change mediates GFP-PieA recruitment to the vacuole.

### Deletion analysis reveals PieA domains important for recruitment to mature vacuoles containing *L. pneumophila*

**Figure 5:**
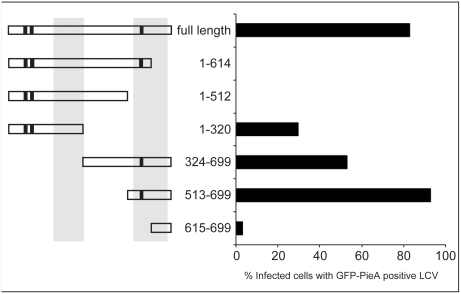
Deletion analysis of the PieA protein reveals a C-terminal domain sufficient for the recruitment of PieA to vacuoles containing *L. pneumophila*. CHO FcγRII cells transiently expressing the indicated PieA deletion constructs N-terminally fused to GFP were infected with wild-type *L. pneumophila*. Cells were fixed seven hours post infection and scored for the percent of PieA-expressing infected cells where PieA was detected decorating vacuoles containing *L. pneumophila*. Values are means from two independent experiments in which 30 vacuoles were scored for each condition. A schematic illustration of the PieA protein sequence shows the different truncation constructs, as well as the predicted coiled-coil domains (black bars) and the identified vacuole recruitment domain (VRD - grey bars). PieA C-terminal residues 513–699 were found to be sufficient for mediating the recruitment of the protein to vacuoles containing *L. pneumophila*.

Deletion derivatives were constructed to identify amino acid regions within PieA that are important for interaction of the protein with vacuoles containing *L. pneumophila*. All of the eukaryotic expression plasmids encoding the GFP-PieA deletion constructs described in Figure 5 produced similar levels of protein after transfection (data not shown). The recruitment of each PieA deletion derivative to vacuoles containing *L. pneumophila* was measured by fluorescence microscopy after infection (Figure 5). These data show that a GFP fusion protein containing C-terminal residues 513–699 of PieA was recruited to vacuoles containing *L. pneumophila* as efficiently as the full-length GFP-PieA protein. This region of PieA was designated the Vacuole Recruitment Domain (VRD). The GFP-PieA(1–512) protein, having the C-terminal VRD deleted, did not co-localize with vacuoles containing *L. pneumophila*, which indicates that the VRD is both sufficient and important for vacuole recruitment of PieA. A central region of PieA was found to have homology to the C-terminal region containing the VRD (Figure 5, grey bars). Although the internal region with similarity to the VRD region could not mediate recruitment of GFP-PieA(1–512) and GFP-PieA(1–614) to the vacuole, production of GFP-PieA(1–320) resulted in localization of the protein to the vacuole at low efficiency. Thus, there are discrete regions in PieA that can target this effector protein to vacuoles containing *L. pneumophila*.

### *In vitro* binding of PieA to isolated vacuoles containing *L. pneumophila*

**Figure 6:**
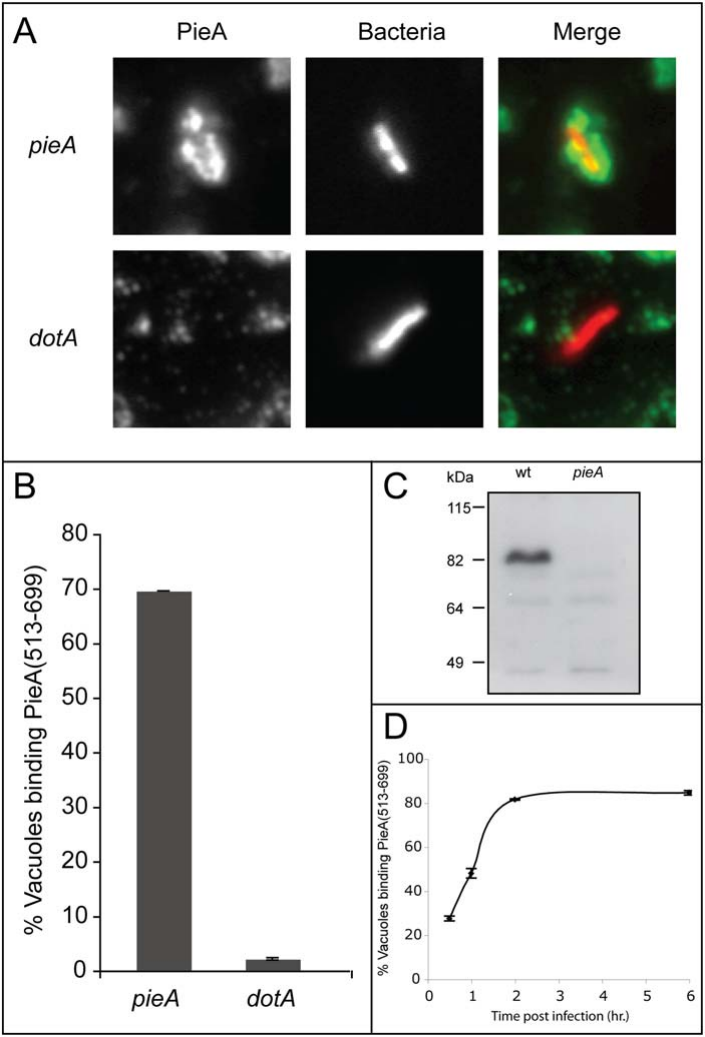
PieA binds *in vitro* to vacuoles containing *L. pneumophila* with an intact Dot/Icm system, but not to vacuoles containing a *dotA* mutant. *L. pneumophila* vacuoles were isolated from U937 macrophage-like cells infected with either a *pieA* mutant or a *dotA* mutant strain expressing the fluorescent protein DsRed. (A) Representative epifluorescent micrographs of *L. pneumophila* containing vacuoles that were incubated with purified PieA(513–699) protein. Bound protein (PieA) was detected using a polyclonal antibody directed against PieA and was found on vacuoles containing the *pieA* strain, but not on vacuoles containing the Dot/Icm deficient strain *dotA*. (B) *L. pneumophila* containing vacuoles were scored to quantify the percent of vacuoles that bound to PieA. Values are means±standard error of mean for three independent experiments in which 300 vacuoles were scored for each condition. (C) An immunoblot probed with an αPieA antibody. A specific band corresponding to a protein with the same molecular weight predicted for the *pieA* product was detected in whole-cell lysates from wild-type *L. pneumophila*. The αPieA–reactive product was not detected in lysates isolated from *pieA* mutant *L. pneumophila*. The positions of molecular weight standards (kDa) are indicated to the left of the immunoblot. (D) PieA binding to vacuoles isolated from U937 macrophage-like cells at different times post infection with wild-type *L. pneumophila*. Values are means±standard error of mean for three independent experiments. PieA binding reaches a maximum in vacuoles isolated two hours post infection.

To better characterize the binding of PieA to vacuoles, a cell-free system was established. Vacuoles containing an *L. pneumophila pieA* mutant were isolated from infected U937 macrophage-like cells and immobilized on glass coverslips. Purified PieA(513–699) protein was incubated with vacuoles. PieA association was determined by immunofluorescence microscopy following staining of vacuoles with an αPieA antibody (Figure 6). Fluorescence microscopy clearly revealed PieA(513–699) protein surrounding vacuoles containing *L. pneumophila*. These data show that the C-terminal VRD region in PieA defined *in vivo* mediates protein binding to isolated vacuoles *in vitro*. Vacuoles containing Δ*dotA* mutant bacteria were used to determine whether *in vitro* binding of PieA to vacuoles containing *L. pneumophila* was dependent on Dot/Icm-mediated alterations to the organelle (Figure 6A and 6B). There was no detectable binding of PieA(513–699) to vacuoles containing Δ*dotA* bacteria. Thus, Dot/Icm-dependent modifications to vacuoles containing *L. pneumophila* are required for PieA binding both *in vivo* and *in vitro*. The efficiency of PieA binding to vacuoles varied depending on the time the vacuoles were isolated after infection. Vacuoles isolated later in infection achieved a higher level of PieA binding. Optimal PieA binding was obtained for vacuoles isolated from cells that were infected for at least two hours (Figure 6D). PieA binding to vacuoles containing the *icmS icmW* double mutant was also tested because the translocation of multiple effector proteins is abrogated in this mutant [26],[41]. PieA binding to vacuoles containing the *icmS*, *icmW* mutant was impaired, with only 50% binding activity relative to wild-type-containing vacuoles (Figure S4). Taken together, these data indicate that Dot/Icm-dependent maturation events mediated by effectors, requiring IcmSW function for translocation, enable the efficient binding of PieA to vacuoles containing *L. pneumophila*.

### Protein on the cytoplasmic surface of the vacuole containing *L. pneumophila* is required for PieA binding

**Figure 7:**
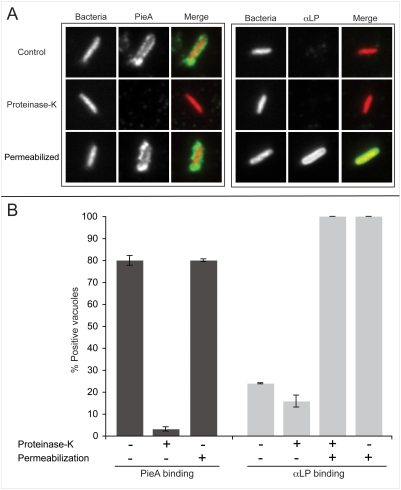
PieA binds to the cytoplasmic face of vacuoles containing *L. pneumophila*, in a mechanism dependent upon protein–protein interaction. Representative epifluorescence micrographs of *L. pneumophila* vacuoles isolated from U937 macrophage-like cells infected with a *pieA* mutant strain expressing the fluorescent protein DsRed. (A) *L. pneumophila* containing vacuoles that were treated with PK to eliminate surface exposed protein epitopes (Proteinase-K), lost their ability to bind PieA (PieA), as compared to vacuoles treated under the same conditions without PK (Control). PK treated vacuoles remained intact and their content remained inaccessible to an antibody directed against *L. pneumophila* (αLP), unless the vacuoles were first permeabilized using cold methanol (Permeabilzed). (B) The isolated vacuoles were scored to quantify the percent of vacuoles that bound to PieA(513–699) (PieA) or stained positive for *L. pneumophila* (αLP). Values are means±standard error of mean for three independent experiments in which 300 vacuoles were scored for each condition.

The *in vitro* assay was used to determine whether there is a protein determinant on the vacuole containing *L. pneumophila* that is important for PieA binding. Proteins on the surface of vacuole containing *L. pneumophila* were digested with Proteinase-K (PK) prior to incubation with purified PieA(513–699). Treatment of vacuoles with PK greatly reduced PieA(513–699) binding (Figure 7A, left panel). PieA(513–699) was associated with 79±1 percent of vacuoles in the untreated control reactions compared to less than 3 percent of the vacuoles that were digested with PK (Figure 7B). Because PK treatment might disrupt the vacuole membrane surrounding *L. pneumophila*, membrane integrity was assessed after PK digestion by staining isolated vacuoles with an antibody that binds to LPS on the bacterial surface (αLP). The percentage of untreated vacuoles that stained positive using the αLP antibody (24±0.6) did not increase after PK treatment (16±3). All of the vacuoles stained positive with the αLP antibody when the surrounding membrane was permeabilized with methanol before antibody incubation (Figure 7A and 7B). Thus, PK treatment did not affect the integrity of the membrane surrounding isolated vacuoles containing *L. pneumophila*, indicating that the inability of PieA to bind to these vacuoles is caused by digestion of a protein exposed on the cytoplasmic surface of the organelle.

## Discussion

Genomic plasticity has been demonstrated for the *L. pneumophila* genome in work comparing the genomes of two serogroup1 isolates Lens and Paris [18]. More than ten percent of the genes in each genome were found to be strain specific [18]. How genomic plasticity might impact the repertoire of translocated effectors and bacterial phenotypes has not been investigated. It is possible that *L. pneumophila* strains have evolved in association with different protozoan hosts and that the predominant host species encountered by an individual strain in nature will impact the acquisition and maintenance of genes encoding translocated effectors. In support of this hypothesis, we demonstrate in this study a region of genomic plasticity that encodes multiple translocated effectors of the Dot/Icm system, which we called the Pie region.

The Pie region within the strain Philadelphia1 was found to contain genes encoding seven different Dot/Icm substrates. Analysis of the Pie region in other sequenced strains of *L. pneumophila* revealed that the *pieB* gene is found only in the Philadelphia1 genome, *pieE*, *pieF* and *pieG* are present in all four genomes, and *pieA*, *pieC* and *pieD* are present in two or more of the four genomes (Figure 1). *pieA*, *pieB*, *pieC* and *pieD* have a significantly lower GC content than the genomic average. This finding suggests that these *pie* genes were acquired through horizontal gene transfer, from foreign DNA. Because some of the *pie* genes are not present in all *L. pneumophila* genomes it is possible that they were acquired after the strain sub-speciation took place. Another possibility is that the genes were lost from the Lens and Paris genomes due to lower selection pressure for their existence in these strains.

This work clearly illustrates that regions of genomic plasticity in *L. pneumophila* can contain genes encoding Dot/Icm substrates. *L. pneumophila* effectors have been identified using several genetic and biochemical methods [21]–[29], including a systematic search for eukaryotic-like *L. pneumophila* proteins [18]–[20]. Our data suggests that further examination of other regions of genomic plasticity will likely reveal additional *L. pneumophila* effectors that have no obvious homology to eukaryotic proteins, which would include Dot/Icm substrates that arose by convergent evolution of proteins unrelated to the eukaryotic factors they mimic or perturb.

PieA was further investigated because of the unique ability of this protein to bind to vacuoles containing *L. pneumophila*. Data obtained from *in vivo* recruitment and *in vitro* binding of PieA to vacuoles containing *L. pneumophila* indicate that a protein on the cytoplasmic surface of vacuoles is required for PieA interaction. Other requirements for PieA binding were that the bacteria within the vacuoles must have a functional Dot/Icm system and vacuoles must have matured in the host cell for at least one to two hours. These data suggest that accumulation of an effector or effector complex on the vacuole is necessary for PieA recruitment and that it takes roughly two hours to achieve the required concentration of the PieA recruitment-determinant on the vacuole. Alternatively, it is possible that the Dot/Icm system mediates vacuole recruitment of a host protein that is not found on other ER-derived vesicles in the cell, and it is this host determinant that is critical for PieA binding. Importantly, PieA was not recruited to the ER-derived vacuole containing *B. abortus*. These data suggest that PieA recruitment to vacuoles is not a general phenomenon that occurs after invasion of the ER by pathogens with a functional type IV secretion system. Thus, it is likely that PieA recruitment is either mediated directly by a *L. pneumophila* effector or indirectly by an activity mediated by *a L. pneumophila* effector that is not present in *B. abortus*.

The survival of *L. pneumophila* within host cells involves an ordered series of events that are controlled by the Dot/Icm system. Within minutes of infection, the Dot/Icm system stimulates efficient uptake of *L. pneumophila* by the host phagocyte [42], rapidly prevents fusion of endocytic vesicles with the vacuole [43]–[45], and stimulates transport and binding of ER-derived vesicles to the limiting membrane of the vacuole [14],[15]. During replication there is evidence the Dot/Icm system stimulates ubiquitination of protein on the vacuole surface [46], modulates NF-kB activation [47], and interferes with protein translation [48]. At late stages of infection the Dot/Icm system assists in controlling bacterial egress from the spent host cell [32].

There is evidence that *L. pneumophila* effectors involved in controlling distinct cellular processes are spatially and temporally regulated. Transcriptional control of effector protein expression is one mechanism that could account for temporal regulation [28],[29]. Spatial regulation of DrrA on the vacuole has been demonstrated [25], and is mediated by host determinants on the plasma membrane that are presumably lost or modified as vacuoles mature and acquire new membrane from early secretory vesicles. Proteasome-mediated degradation of ubiquitinated effectors [46], and phosphoinositide metabolism on the vacuole membrane [49], are other mechanisms that have been proposed to spatially control *L. pneumophila* effectors. Studies presented here on PieA indicate that specific modifications to vacuoles controlled by other *L. pneumophila* effectors provide spatial and temporal information that is recognized by other effectors. Based on the observation that PieA binding requires a protein determinant on the cytoplasmic face of the vacuole membrane, we speculated that some effectors act as scaffolding proteins that function at specific stages of infection to recruit and retain a subset of effectors that have biochemical functions important for stage specific maturation events. Future studies will focus on determining the proteins on the vacuole membrane that interact with PieA, functioning as determinants important for spatial regulation of PieA. This may lead to the identification of *L. pneumophila* effectors involved in regulating different stages of vacuole maturation.

## Materials and Methods

### Strains and media

All bacterial strains, plasmids and oligonucleotide primers used in this study are listed in Table S1. Unless otherwise noted, chemicals were purchased from Sigma. Bacto-agar, tryptone, and yeast extract were purchased from Difco. *L. pneumophila* strains used in this study were grown on charcoal-yeast extract (CYE) plates as described previously [50],[51]. When needed, chloramphenicol was added to the media at a concentration of 10 µg ml^−1^.

### Cell culture

Primary cells and cell lines were cultured at 37°C in 5% CO2. CHO cells were grown in minimal essential alpha medium (Gibco) containing 10% heat-inactivated fetal bovine serum (FBS). U937 cells were grown in RPMI-1640+10% FBS. The cells were activated with PMA for 48 h and replated in 6-well tissue culture dishes at a concentration of 3×10^6^ per well before infection with *L. pneumophila*. Bone-marrow derived macrophages were cultured from female A/J mice as described previously [52]. HeLa cells (ATCC clone CCL-2) were cultured in Dulbecco's-Modified Eagle Medium (DMEM) supplemented with 10% fetal calf serum (FCS) and 2 mM L-glutamine.

### Construction of isogenic *pie* deletion strains

All *L. pneumophila* mutants used in this study were derived from the wild-type strain Lp01. Gene deletions were introduced onto the chromosome of *L. pneumophila* by allelic exchange as described previously [53]. Deletion alleles of the *pie* genes were constructed using polymerase chain reaction (PCR) to generate DNA fragments encoding regions of flanking homology that were immediately 5′ to the start codon and 3′ to the termination codon of each gene, or set of consecutive genes. The primers used were SN76–SN77 and SN78–SN79 for the ppeA–ppeB deletion allele, SN152–SN153 and SN154–SN155 for the *pieE* deletion allele, SN129–SN149 and SN150–SN151 for the *pieA–pieD* deletion allele, SN133–SN134 and SN135–SN136 for deletion allele lpg1975–*pieG*. For each deletion allele, the 5′ and 3′ PCR products were joined by recombinant PCR and the final product was digested with the enzymes indicated in Table S1, and ligated into the gene replacement vector pSR47s digested similarly, creating the plasmids listed in Table S1. Deletion mutant strain SN178 was created by progressive gene deletions using the above replacement vectors. For the insertional-inactivation of *pie* genes, primers SN72–SN73 were used for the *ppgA* allele, and primers SN74–SN75 for the *pieA* allele. The resulting PCR products were ligated into suicide vector pSR47 creating plasmids pSN44 (*ppgA*) and pSN45 (*pieA*). Mutant strain SN179 was constructed by integrating plasmid pSN44 into the genome of strain SN178. Mutant strain SN122 was constructed by integrating plasmid pSN45 into the genome of strain Lp01.

### Plasmid construction

The following sets of primers were used for cloning potential effector proteins: SN34 and SN35 for PieC, SN44 and SN45 for PieD, SN46 and SN47 for PieG, SN48 and SN49 for PieE, SN50 and SN51 for PieA, SN141 and SN142 for PieB, SN145 and SN146 for lpg1975, SN166 and SN167 for PpeA, SN168 and SN169 for PpeB, SN170 and SN171 for PpgA, SN175 and SN176 for PieF, SN143 and SN144 for lpg1968, SN177 and SN178 for lpg1973. The resulting PCR products were digested with the appropriate enzymes (see Table S1), and ligated to both vector pCya digested BamHI/PstI, or vector pEGFP-C2 digested BglII/PstI. The resulting pCya derived vectors encode for fusion proteins consisting of an amino-terminal M45 epitope tag, followed by amino acid residues 2–399 of the *B. pertussis* CyaA enzyme followed by the indicated *L. pneumophila* protein. Expression of the Cya fusion proteins is driven by the *icmR* promoter located upstream. The resulting pEGFP derived vectors encode for fusion proteins consisting of EGFP followed by the indicated *L. pneumophila* protein. The names and content of the generated plasmids are listed in Table S1. For the construction of 6His-tagged PieA primers SN91 and SN99 (PieA) or SN147 and SN148 (PieA513–699) were used. The resulting PCR products were digested with NdeI and BamHI, and ligated with the pET15b vector digested similarly, resulting in plasmids pSN63 and pSN73 respectively. For the construction of a DsRed-Express *L. pneumophila* expression vector, primers DsRed T1 Fwd and DsRed T1 Rev were used to amplify the DsRed-Express coding sequence from plasmid pDsRed-Express. The PCR product was digested with BamHI and HindIII, and ligated with plasmid pMMB207 digested similarly, resulting in plasmid pEMC22 encoding for the DsRed-Express protein under the control of the *tac* and *icmR* promoters located upstream. For the construction of PieA C-terminal deletion constructs fused to GFP, plasmid pSN39 was digested with NheI and HindIII, NheI and EcoRI, or NheI and SacI to obtain DNA fragments encoding for GFP-PieA(1–323), GFP-PieA(1–512) and GFP-PieA(1–614) respectively. The resulting fragments were ligated with plasmid pEGFP-C2 digested similarly, creating plasmids pSN56, pSN55 and pSN54 respectively. For the construction of PieA N-terminal deletion constructs fused to GFP, plasmid pSN39 was digested with HindIII and DraIII, EcoRI and DraIII, or SacI and DraIII to obtain DNA fragments encoding PieA(324–699), PieA(513–699) and PieA(615–699), respectively. The resulting fragments were ligated with plasmid pEGFP-C1 (for PieA(324–699) and PieA(513–699)), or with plasmid pEGFP-C3 (for PieA(615–699)) digested similarly, creating plasmids pSN59, pSN60 and pSN61 respectively.

### Translocation assay

Translocation of potential substrates into host cells was assayed using the Cya fusion approach described previously [26],[33]. Briefly, a stable CHO cell line producing Fc*γ*RII [54] was used, cells were plated at 1×10^5^ cells per well in a 24-well tissue-culture-treated dish, and infected on the next day with the desired *L. pneumophila* strain carrying plasmids pSN24 (PieA), pSN65 (PieB), pSN20 (PieC), pSN27 (PieD), pSN25 (PieE), pSN87 (PieF), pSN28 (PieG), pSN81 (PpeA), pSN82 (PpeB), pSN83 (PpgA), pSN66 (lpg1968), pSN88 (lpg1973) or pSN67 (lpg1975) expressing the Cya fused to the gene of interest. The cells were infected at a multiplicity of infection (MOI) of 30, and then spun five minutes at 1000 rpm to initiate contact and synchronize the infection. Infected cells were incubated for one hour at 37°C with 5% CO_2_. Cells were washed three times in ice-cold phosphate-buffered saline (PBS) and lysed in cold buffer containing 50 mM HCl and 0.1% triton x-100 for 30 minutes at 4°C. The lysates were boiled for five minutes, and neutralized with 30 mM NaOH. Levels of cAMP were determined using the cAMP Biotrak enzymeimmunoassay (EIA) system (Amersham Biosciences).

### Intracellular growth assays

Intracellular growth assays were conducted in *A. castellanii* (ATCC strain 30234) or in bone marrow derived murine macrophages, as described previously [26],[55].

### *B. abortus* infection

A derivative of *B. abortus* strain 2308 constitutively expressing monomeric DsRed (DsRedm) was generated as described [56]. HeLa cells seeded on 12 mm glass coverslips in 24-well plates were transfected using the FuGene 6™ transfection reagent (Roche) to express GFP-PieA 24 hours before infections. For infections, bacteria grown to late log phase in Tryptic Soy broth were diluted in complete medium and added to chilled cells at a theoretical multiplicity of infection (MOI) of 500. Bacteria were centrifuged onto cells at 400× g for 10 minutes at 4°C, and infected cells were incubated for 30 minutes at 37°C under 7% CO2 atmosphere following a rapid warm up in a 37°C water bath to synchronize bacterial entry. Infected cells were then washed five times with DMEM to remove extracellular bacteria, incubated for an additional 60 minutes in complete medium before medium containing 100 µg/ml gentamicin was added for 90 min to kill extracellular bacteria. Thereafter, infected cells were maintained in gentamicin-free medium. At 24 hours post infection infected cells were washed three times with PBS, fixed with 3% PFA.

### Fluorescence microscopy

For localization of GFP-tagged effectors, CHO FcγRII cells were plated on 12-mm glass coverslips in 24-well tissue culture plates at a density of 10^4^ cells per well. FuGene 6™ (Roche) was used to transfect the cells with plasmids pSN39 (GFP-PieA), pSN69 (GFP-PieB), pSN30 (GFP-PieC), pSN35 (GFP-PieD), pSN37 (GFP-PieE) or pSN33 (GFP-PieG). After 18 hours of expression cells were either directly fixed using 2% paraformaldehyde (PFA), or first infected with wild-type *L. pneumophila* strain Lp01 at an MOI of 30, and fixed seven hours post infection. For PieA deletion analysis, cells were seeded similarly, and transfected with plasmids pSN39 (GFP-PieA), pSN54 (GFP-PieA(1–614)), pSN55 (GFP-PieA(1–512)), pSN56 (GFP-PieA(1–323)), pSN59 (GFP-PieA(324–699)), pSN60 (GFP-PieA(513–699)) or pSN61 (GFP-PieA(615–699)). After 18 hours of expression cells were infected with wild-type *L. pneumophila* strain Lp01 at an MOI of 20. Seven hours post infection cells were washed with PBS supplemented with 0.9 mM CaCl_2_ and 1 mM MgCl_2_ and pre-permeabilized using 0.1% saponin in pipes buffer (80 mM pipes, 5 mM EGTA, 1 mM MgCl_2_, pH 6.8) for five minutes before fixing with 2% PFA. For detection of host and bacterial DNA, cells were stained with 4,6-diamidino-2-phenylindole (DAPI) for ten minutes at 25°C. For Lamp1 detection cells were permeabilized with 0.1% saponin (Sigma), and stained with UH1 mouse anti-hamster Lamp1 monoclonal antibody (Developmental Studies Hybridoma Bank), followed by secondary TexasRed anti–mouse IgG (Invitrogen). For KDEL detection, cells were first permeabilized using cold methanol, and stained with a mouse anti-KDEL monoclonal antibody (Stressgene) followed by secondary TexasRed anti–mouse IgG (Invitrogen). Polyclonal antibodies against PieA were produced at Pocono Rabbit Farm and Laboratory (Canadensis, Pennsylvania) using affinity purified histidine-tagged protein as antigen to immunize rabbits. Digital images were acquired with a Nikon TE300 microscope using a 100× 1.4 N.A objective lens and a Hamamatsu ORCA-ER camera controlled by IP Lab software. Images were exported as TIFF files and labeled in Adobe Photoshop.

For *B. abortus* infection experiments samples were blocked and permeabilized in 10% horse serum, 0.1% saponin in PBS for 30 min at room temperature. Cells were labeled using mouse anti-human LAMP-1 clone H4A3 antibody (developed by J. T. August and obtained from the Developmental Studies Hybridoma Bank developed under the auspices of the NICHD and maintained by The University of Iowa, Department of Biological Sciences, Iowa City, Iowa) or rabbit polyclonal anti-calreticulin antibodies (Affinity BioReagents) for 45 min at room temperature. Bound antibodies were detected using Cyanin 5-conjugated donkey anti-mouse antibodies (Jackson ImmunoResearch Laboratories). Samples were observed and imaged on a Carl Zeiss LSM 510 confocal laser-scanning microscope. Confocal images of 1024×1024 pixels were acquired as projections of 3 consecutive slices with a 0.38 µm step and assembled using Adobe Photoshop CS. For endogenous PieA detection vacuoles were isolated from U937 macrophage-like cells as described below and stained using rabbit polyclonal αPieA antiserum at 1∶100 dilution, followed by secondary FITC anti–mouse IgG (Invitrogen).

### *In vitro* PieA binding assay

U937 macrophage-like cells were seeded into 6-well tissue culture plates at a density of 3×10^6^ macrophages per well. The next day, the cells were infected with the indicated *L. pneumophila* strain harboring plasmid pEMC22, that were plate grown for two days in the presence of 1 mM Isopropyl β-D-1-thiogalactopyranoside (IPTG) to induce the expression of the fluorescent protein DsRed-Express. The bacteria were added to the cells at an MOI of 5 in the presence of 1 mM IPTG, and spun five minutes at 1000 rpm to initiate contact and synchronize the infection. One hour post-infection extracellular bacteria were removed by washing each well three times with warm PBS. Wells were refreshed with tissue culture medium containing 1 mM IPTG and incubated at 37°C for an additional hour. Next, the cells were placed on ice, and the wells were washed with cold PBS, before cells were lifted using a cell scraper into 1 ml cold homogenization buffer (H.B) containing 250 mM sucrose, complete protease inhibitor cocktail (Roche), and 20 mM Hepes pH 7.2. The cells were homogenized using a ball-bearing homogenizer, and the cell homogenate was spun for three minutes at 1500 rpm to sediment cell nuclei and unbroken cells. The post-nuclear supernatant (PNS) was diluted 1∶5 in cold H.B. and spun onto poly-L-lysine coated 12-mm glass coverslips in 24-well tissue culture plates. The PNS was fixed by the addition of PFA to 2% for 20 minutes at 25°C. To test for PieA binding, coverslips were incubated for one hour at 4°C with a blocking solution containing 50 mM ammonium sulfate and 2% goat serum, supplemented with 2 µg/ml of affinity purified 6His-PieA(513–699) protein. To remove unbound protein, the coverslips were washed three times in PBS. For detection of bound protein coverslips were stained with αPieA polyclonal antiserum at 1∶500 dilution, followed by secondary FITC anti–rabbit IgG (Invitrogen). Where indicated, vacuoles were permeabilized for 10 s with ice-cold methanol. The integrity of the vacuolar membranes was tested using polyclonal antiserum specific for *L. pneumophila* serogroup1 (αLP), and FITC-conjugated anti-rabbit IgG (Invitrogen). Where indicated, coverslips were first treated with PK by incubating them for two hours at 37°C in PBS to which PK was added to a final concentration of 10 µg/ml. The reaction was stopped by washing the coverslips three times in PBS, and then incubating them for 10 minutes in 1 mM PMSF in PBS, and finally washing three times with PBS. Control samples were treated similarly, but without the addition of PK.

### Reverse transcription–PCR (RT–PCR)

RNA was isolated from broth-grown *L. pneumophila* using the TRIzol Max bacterial RNA isolation kit (Invitrogen). The RNA was digested with DNase Using an On-Column DNase digestion kit (Qiagen). RT-PCR was performed in two steps. First strand synthesis was performed using superscript II reverse transcriptase kit (Invitrogen) using 5 µg of total RNA and random primers (Invitrogen), and included a negative control reaction without reverse transcriptase. The PCR was preformed using taq-polymerase (Invitrogen) with gene-specific primers, and a 1∶50 dilution of the first strand mix as template.

### Accession numbers

The NCBI accession numbers for the proteins discussed in this paper are *L. pneumophila* PieA (YP_095979), PieB (YP_095980), PieC (YP_095981), PieD (YP_095982), PieE (YP_095985), PpeA (YP_095728), PpeB (YP_095729), PieF (YP_095988), PiG (YP_095992) and PpgA (YP_096236).

## Supporting Information

Table S1 Strains, plasmids and primers
(0.10 MB PDF)

Figure S1 The protein encoded by lpg1965 is translocated into host cells in a Dot/Icm dependent manner. CHO cells were infected, at a multiplicity of infection (MOI) of 30, with *L. pneumophila* strains harboring plasmid pSN20 expressing the Cya-lpg1965 fusion proteins, under the *icmR* promoter. One hour after infection cells were lysed and cAMP was extracted and quantified as described under Materials and Methods. To test for Dot/Icm dependency, translocation was assayed both in cells infected with the wild-type strain Lp01 (wt) as well as with the *dotA* mutant CR58 (*dotA*). To test for IcmS and IcmW dependency, translocation was assayed in cells infected with double mutant CR503 lacking both *icmS* and *icmW* (*icmW icmS*). Levels of cAMP were also determined in uninfected cells (uninfected). Each bar represents the mean cAMP value obtained from triplicate wells±standard deviation.
(9.78 MB TIF)

Figure S2 The *pie* genes encoded in the Pie genomic region are expressed. RT-PCR analysis was performed using RNA isolated from broth-grown *L. pneumophila*. Reactions were carried out using primers specific for the genes *pieA*, *pieB*, *pieC*, *pieD*, *pieE*, *pieF* and *pieG*. PCRs in which no reverse transcriptase was added during the first-strand synthesis step were conducted to control for residual DNA (no RT).
(2.37 MB TIF)

Figure S3 Translocation of all Pie proteins is dependent upon the IcmS-IcmW complex. Translocation was assayed as described earlier in cells infected with wild-type *L. pneumophila* or with a double mutant CR503 lacking both *icmS* and *icmW*. Translocation efficiencies were calculated by dividing cAMP levels measured for the mutant strain by the cAMP levels measured in a parallel infection using wild-type *L. pneumophila* producing the indicated Cya fusion protein and multiplying by 100 to give percent translocation (relative to wild-type). All infections were performed in triplicate with a standard deviation of less than 10% of the average.
(9.32 MB TIF)

Figure S4 PieA binding to vacuoles containing *L. pneumophila* is impaired in the absence of the IcmS-IcmW complex. *L. pneumophila* vacuoles were isolated from U937 macrophage-like cells infected with either wild-type bacteria (wt), or a mutant strain lacking the *icmS* and *icmW* genes (*icmS icmW*). Vacuoles were incubated with purified PieA(513–699) protein and bound protein was detected using a polyclonal antibody directed against PieA. *L. pneumophila* containing vacuoles were scored to quantify the percent of vacuoles that bound to PieA. Values are means±standard-error of mean for three independent experiments.
(7.89 MB TIF)
