# Supplementary material for: A Legionella pneumophila Effector Protein Encoded in a Region of Genomic Plasticity Binds to Dot/Icm-Modified Vacuoles
Source: PLoS Pathog. 2009 Jan 23;5(1):e1000278. doi: 10.1371/journal.ppat.1000278 (PMC2621349; doi:10.1371/journal.ppat.1000278)
Supplement: Table S1 — Strains, plasmids and primers (0.10 MB PDF) [file ppat.1000278.s001.pdf]

Supporting Table S1. Strains, plasmids and primers.

L. pneumophila strains

Name	Genotype	Reference
Lp01	*L.pneumophila* serogroup 1, strain Lp01 *rpsL*	*[1]*
CR58	Lp01 *rpsL ΔdotA*	*[2]*
CR503	*ΔicmS ΔicmW rpsL*	*[3]*
SN178	Lp01 *rpsL ΔppeA-ppeB ΔpieE ΔpieA-pieD Δlpg1975-pieG*	*This study*
SN179	Lp01 *rpsL ΔppeA-ppeB ΔpieE ΔpieA-pieD Δlpg1975-pieG ppgA::kan^r*	*This study*
SN122	Lp01 *rpsL pieA::kan^r*	*This study*

Plasmids

Name	Important properties	Marker	Reference
pCya	pMMB207 encoding M45-Cya	Cm	[4]
pDsRed-Express	Prokaryotic expression vector encoding DsRed-Express	Amp	Clontech
pEGFP-C1	Eukaryotic expression vector encoding EGFP	Kan	Clontech
pEGFP-C2	Eukaryotic expression vector encoding EGFP	Kan	Clontech
pEGFP-C3	Eukaryotic expression vector encoding EGFP	Kan	Clontech
pEMC22	pMMB207 encoding DsRed-Express	Cm	This study
pET15b	6His-tag expression vector	Amp	Novagen
pMMB207	cloning vector derived from RSF1010	Cm	[5]
pSN20	pCya encoding the Cya-PieC fusion protein	Cm	This study
pSN24	pCya encoding the Cya-PieA fusion protein	Cm	This study
pSN25	pCya encoding the Cya-PieE fusion protein	Cm	This study
pSN27	pCya encoding the Cya-PieD fusion protein	Cm	This study
pSN28	pCya encoding the Cya-PieG fusion protein	Cm	This study
pSN30	pEGFP ancoding the GFP-PieC fusion protein	Kan	This study
pSN33	pEGFP ancoding the GFP-PieG fusion protein	Kan	This study
pSN35	pEGFP ancoding the GFP-PieD fusion protein	Kan	This study
pSN37	pEGFP ancoding the GFP-PieE fusion protein	Kan	This study
pSN39	pEGFP ancoding the GFP-PieA fusion protein	Kan	This study
pSN45	pSR47 for insertional inactivation of *pieA*	Kan	This study
pSN53	pSR47s for *ppeA-ppeB* deletion	Kan	This study
pSN54	pEGFP ancoding the GFP-PieA(1-614) fusion protein	Kan	This study
pSN55	pEGFP ancoding the GFP-PieA(1-512) fusion protein	Kan	This study
pSN56	pEGFP ancoding the GFP-PieA(1-323) fusion protein	Kan	This study
pSN59	pEGFP ancoding the GFP-PieA(324-699) fusion protein	Kan	This study
pSN60	pEGFP ancoding the GFP-PieA(513-699) fusion protein	Kan	This study
pSN61	pEGFP ancoding the GFP-PieA(615-699) fusion protein	Kan	This study
pSN63	pET15b encoding 6His-PieA	Amp	This study
pSN64	pSR47s for *lpg1975-pieG* deletion	Kan	This study
pSN65	pCya encoding the Cya-PieB fusion protein	Cm	This study
pSN66	pCya encoding the Cya-lpg1968 fusion protein	Cm	This study
pSN67	pCya encoding the Cya-lpg1975 fusion protein	Cm	This study
pSN69	pEGFP ancoding the GFP-PieB fusion protein	Kan	This study
pSN69	pSR47 for insertional inactivation of *ppgA*	Kan	This study
pSN71	pSR47s for *pieE* deletion	Kan	This study
pSN72	pSR47s for *pieA-pieD* deletion	Kan	This study
pSN73	pET15b encoding 6His-PieA(513-699)	Amp	This study
pSN81	pCya encoding the Cya-PpeA fusion protein	Cm	This study
pSN82	pCya encoding the Cya-PpeB fusion protein	Cm	This study
pSN83	pCya encoding the Cya-PpgA fusion protein	Cm	This study
pSN87	pCya encoding the Cya-PieF fusion protein	Cm	This study
pSN88	pCya encoding the Cya-lpg1973 fusion protein	Cm	This study
pSR47	Suicide vector	Kan	[6]
pSR47s	Gene replacement vector	Kan	[6]

Primers		
Name	Sequence	Restriction site used
DsRed T1 Fwd	CG GAT CCC AGG AGT GGT AAT AAT GGC CTC CTC CGA GGA CGT C	BamHI
DsRed T1 Rev	GGG AAG CTT CTA CAG GAA CAG GTG GTG GCG G	HindIII
SN34	CGAGATCTTTATGCTGAAATTTTACTCAGA	BglII
SN35	GCGCTGCAGTTATTTATTCTCTGGAGTGA	PstI
SN44	GCGGAATTCCGGATCCTTATGATAAAGGACATTCTC	BamHI
SN45	CGCCTCGAGCTGCAGTTAGTTTCTGCAAAACCC	PstI
SN46	GCGGAATTCCGGATCCTTATGAAAACAGTAAAACAA	BamHI
SN47	CGCCTCGAGCTGCAGTCATAACAAATTGCATGG	PstI
SN48	GCGGAATTCCGGATCCTTATGGAGTCTGTATTTTCA	BamHI
SN49	CGCCAATTCTGCAGTTAGTAGCCGTAATTTAC	PstI,
SN50	GCGCTCGAGTGGATCCTTATGCAAGAAAAAATTATC	BamHI
SN51	CGCCTCGAGCTGCAGCTAGATATTCGTAATTCC	PstI
SN72	GCGCTGCAGACGGTCAATTAGGCTGTGG	PstI
SN73	CGCGGTACCATTTCATTCGGGTGAATGTT	
SN74	GCGCTGCAGACTAATTTATACAATGCTGT	PstI
SN75	GCGGGTACCATAATGCGAGATTTCAATA	KpnI
SN76	CGCTCTAGAAATGGCTTCCAATGACGCG	XbaI
SN77	ACGGCTGGATTTGGCCAAAAACATAATCAC	
SN78	TTTTGGCCAAATCCAGCCGTCCGGCATTAA	
SN79	CCGCGGCCGCGTTAGTACAACAGGGGATTT	NotI
SN91	GCGGGATCCCTAGATATTCGTAATTCC	BamHI
SN99	GCGCATATGCAAGAAAAAATTATC	NdeI
SN129	GCGGCGGCCGCATGCTTCCGCAACTGCTAAT	NotI
SN133	GCGGCGGCCGCCTGAACTGGGGCAATTTGTT	NotI
SN134	AACAAATTGCAACCCACGATCTTTGGAATG	
SN135	ATCGTGGGTTGCAATTTGTTATGAGGTTTAATTGG	
SN136	GCGGAGCTCACAACACCATCCATCCCTGT	SacI
SN141	GCGAGATCTTTATGAAAATTTACAGTATAGTTAATC	BglII
SN142	GCGCTGCAGTTAGGTAAAAGTATAGGATAAATC	PstI
SN143	GCGAGATCTTTATGGGGAAATTTTATATGAGTAAA	BglII
SN144	GCGCTGCAGTCATATGTTGGTTATCACTTTCA	PstI
SN145	GCGAGATCTTTGTGGGTTATAATGAAACCATCT	BglII
SN146	GCGCTGCAGTTATTTGATGCAATTCAAGATGC	PstI
SN147	GCGCATATGAATTCACCACAGAGAATAG	NdeI
SN148	GCGGGATCCTAGATATTCGTAATTCCTTTTC	BamHI
SN149	ATTCCTTTTCTTGAGAATGTCCTTTATCATTTA	
SN150	ACATTCTCAAGAAAAGGAATTACGAATATCTAG	
SN151	GCGTCTAGATGAAACTCTCGCATAACAATTC	XbaI
SN152	GCGGCGGCCGCCTAATGGAAAGTGTTGCTATT	NotI
SN153	TTTACTCTTTCCCTGAAAATACAGACTCCAT	
SN154	ATTTTCAGGGAAAGAGTAAATTACGGCTACTAA	
SN155	CGCTCTAGATTGTTGATAAAACTGAGCCGCA	XbaI
SN166	CGCGGATCCTTATGATTATGTTTTTGGCCAACTGCA	BamHI
SN167	GTGCTGCAGTTACGCTATCTCATTAACTGTT	PstI
SN168	GCCGGATCCTTATGTCACTTGCTACTTATGATG	BamHI
SN169	GTGCTGCAGTTAATGCCGGACGGCTGG	PstI
SN170	GTGGGATCCATGAAAGAACCTCGTGAACTT	BamHI
SN171	GTGCTGCAGTTAGGCGCTTCGTTTGGG	PstI
SN175	GTGGGATCCTTATGAAAAGACTAATTATCTGTAA	BamHI
SN176	GTGCTGCAGTCAAATTTTGTTTATTAAGTTATATG	PstI
SN177	CGCGGATCCTTATGACTAACAAAATCTTAAC	BamHI
SN178	GTGCTGCAGCTAGTGTTTTGTTTTTTTCTTAAT	PstI

References

1. Berger KH, Isberg RR (1993) Two distinct defects in intracellular growth complemented by a single genetic locus in *Legionella pneumophila*. Mol Microbiol 7: 7-19.

2. Zuckman DM, Hung JB, Roy CR (1999) Pore-forming activity is not sufficient for *Legionella pneumophila* phagosome trafficking and intracellular growth. Mol Microbiol 32: 990-1001.
3. Coers J, Kagan JC, Matthews M, Nagai H, Zuckman DM, et al. (2000) Identification of Icm protein complexes that play distinct roles in the biogenesis of an organelle permissive for *Legionella pneumophila* intracellular growth. Mol Microbiol. pp. 719-736.
4. Nagai H, Cambronne ED, Kagan JC, Amor JC, Kahn RA, et al. (2005) A C-terminal translocation signal required for Dot/Icm-dependent delivery of the *Legionella* RalF protein to host cells. Proc Natl Acad Sci U S A 102: 826-831.
5. Morales VM, Backman A, Bagdasarian M (1991) A series of wide-host-range low-copy-number vectors that allow direct screening for recombinants. Gene 97: 39-47.
6. Merriam JJ, Mathur R, Maxfield-Boumil R, Isberg RR (1997) Analysis of the *Legionella pneumophila fliI* gene: intracellular growth of a defined mutant defective for flagellum biosynthesis. Infect Immun 65: 2497-2501.
