# Supplementary figures and images for: A Legionella pneumophila Effector Protein Encoded in a Region of Genomic Plasticity Binds to Dot/Icm-Modified Vacuoles
Source: PLoS Pathog. 2009 Jan 23;5(1):e1000278. doi: 10.1371/journal.ppat.1000278 (PMC2621349; doi:10.1371/journal.ppat.1000278)

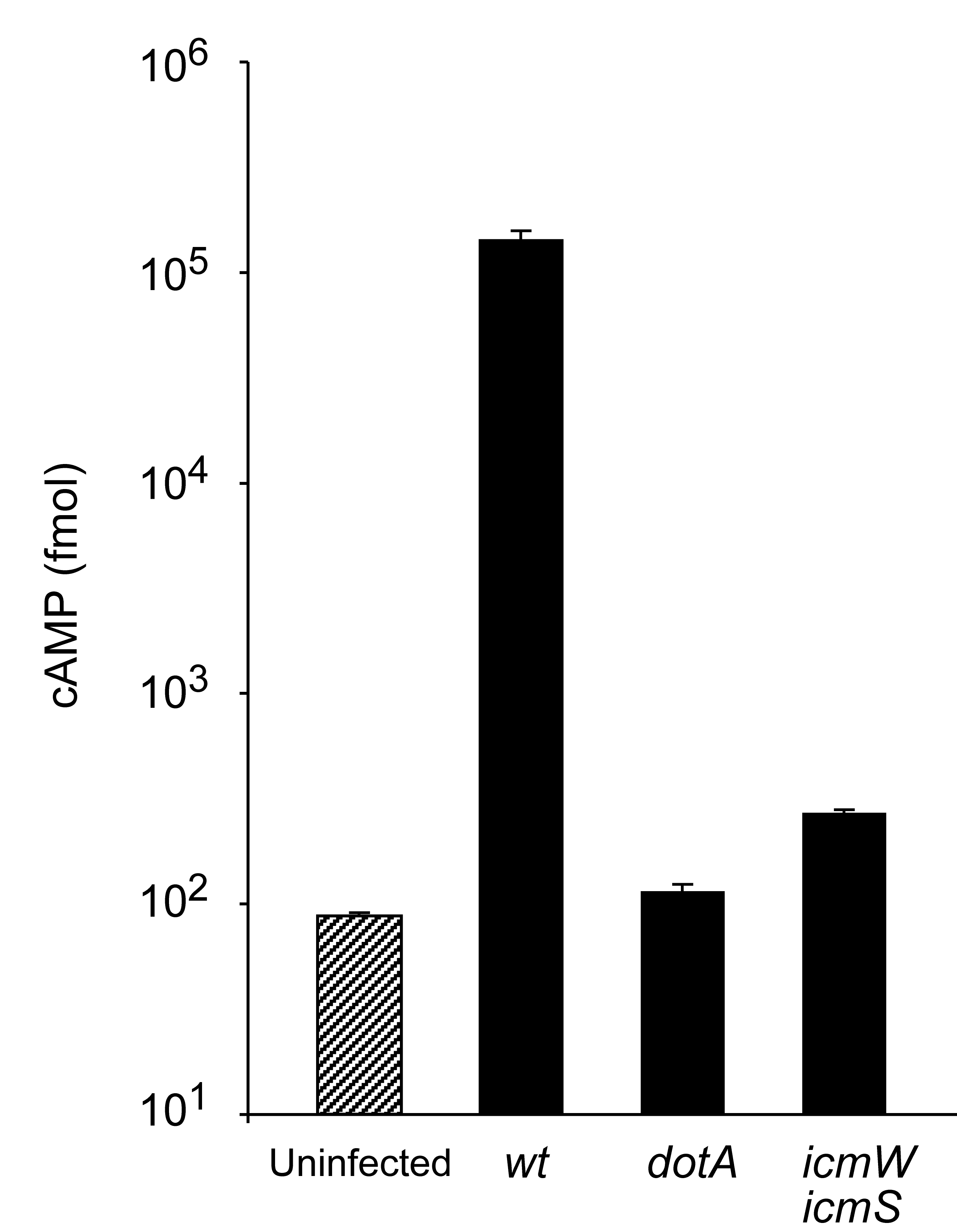

Supplement: Figure S1 — The protein encoded by lpg1965 is translocated into host cells in a Dot/Icm dependent manner. CHO cells were infected, at a multiplicity of infection (MOI) of 30, with L. pneumophila strains harboring plasmid pSN20 expressing the Cya-lpg1965 fusion proteins, under the icmR promoter. One hour after infection cells were lysed and cAMP was extracted and quantified as described under Materials and Methods. To test for Dot/Icm dependency, translocation was assayed both in cells infected with the wild-type strain Lp01 (wt) as well as with the dotA mutant CR58 (dotA). To test for IcmS and IcmW dependency, translocation was assayed in cells infected with double mutant CR503 lacking both icmS and icmW (icmW icmS). Levels of cAMP were also determined in uninfected cells (uninfected). Each bar represents the mean cAMP value obtained from triplicate wells±standard deviation. (9.78 MB TIF) [file ppat.1000278.s002.tif]

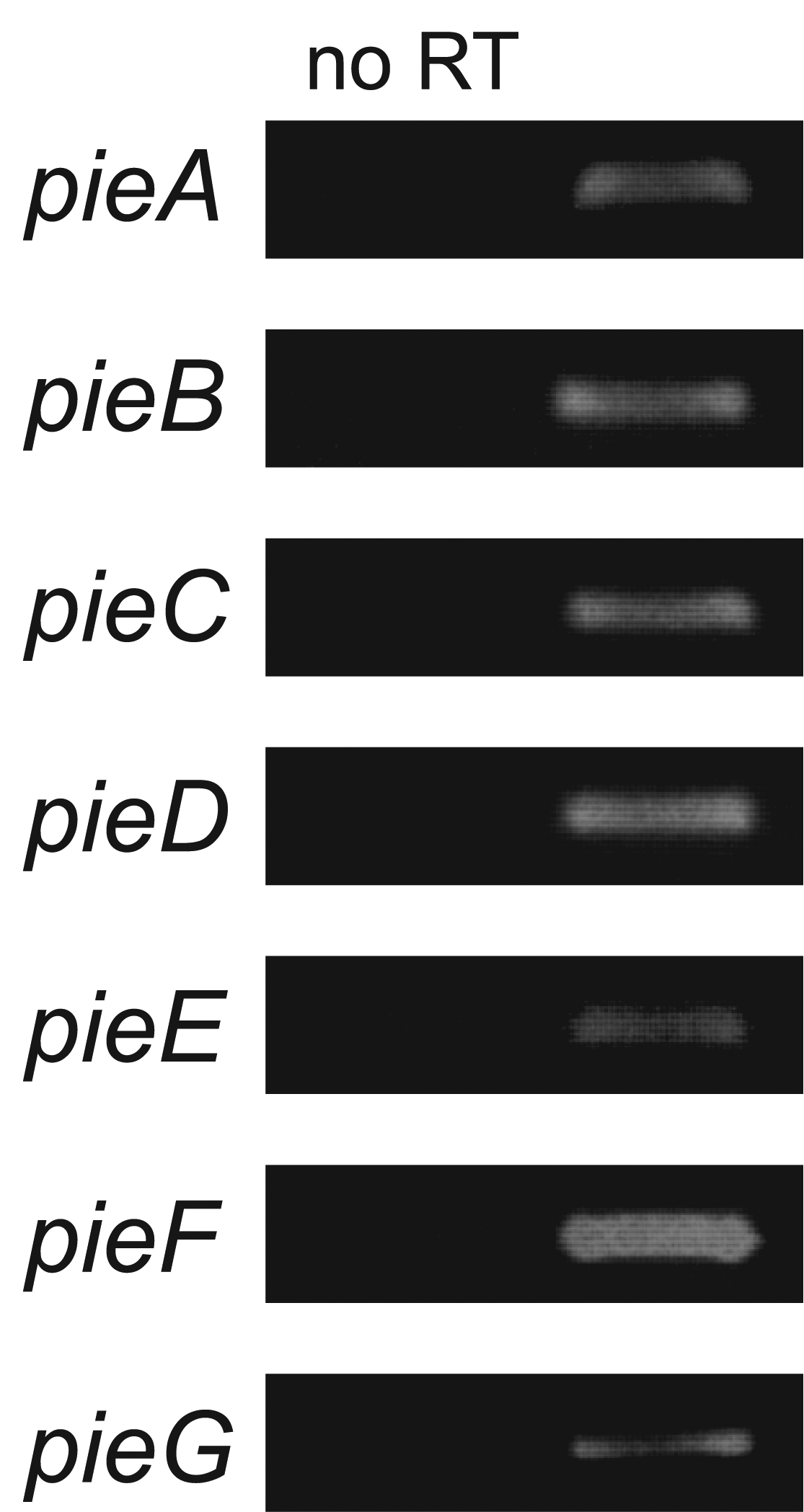

Supplement: Figure S2 — The pie genes encoded in the Pie genomic region are expressed. RT-PCR analysis was performed using RNA isolated from broth-grown L. pneumophila. Reactions were carried out using primers specific for the genes pieA, pieB, pieC, pieD, pieE, pieF and pieG. PCRs in which no reverse transcriptase was added during the first-strand synthesis step were conducted to control for residual DNA (no RT). (2.37 MB TIF) [file ppat.1000278.s003.tif]

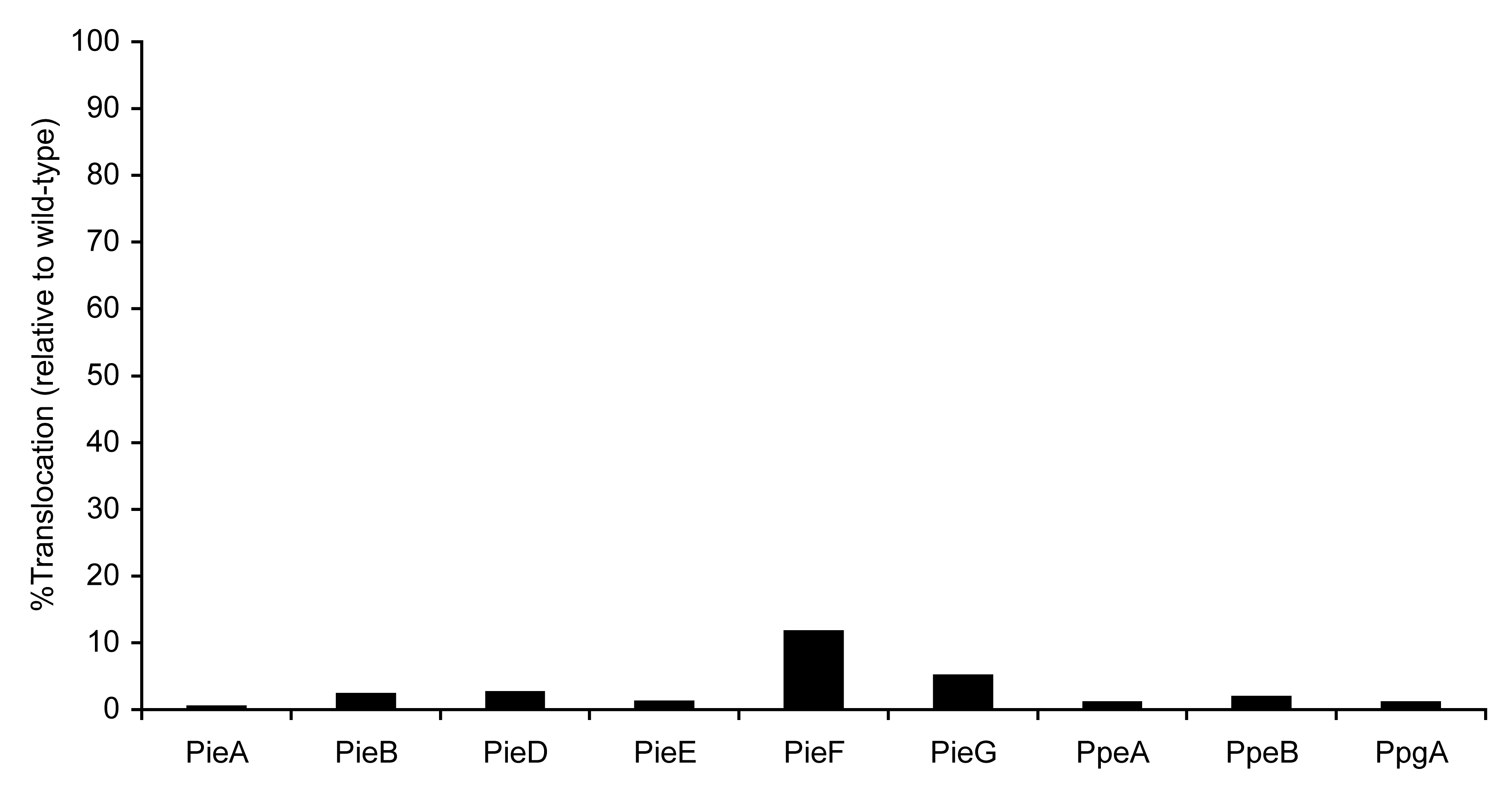

Supplement: Figure S3 — Translocation of all Pie proteins is dependent upon the IcmS-IcmW complex. Translocation was assayed as described earlier in cells infected with wild-type L. pneumophila or with a double mutant CR503 lacking both icmS and icmW. Translocation efficiencies were calculated by dividing cAMP levels measured for the mutant strain by the cAMP levels measured in a parallel infection using wild-type L. pneumophila producing the indicated Cya fusion protein and multiplying by 100 to give percent translocation (relative to wild-type). All infections were performed in triplicate with a standard deviation of less than 10% of the average. (9.32 MB TIF) [file ppat.1000278.s004.tif]

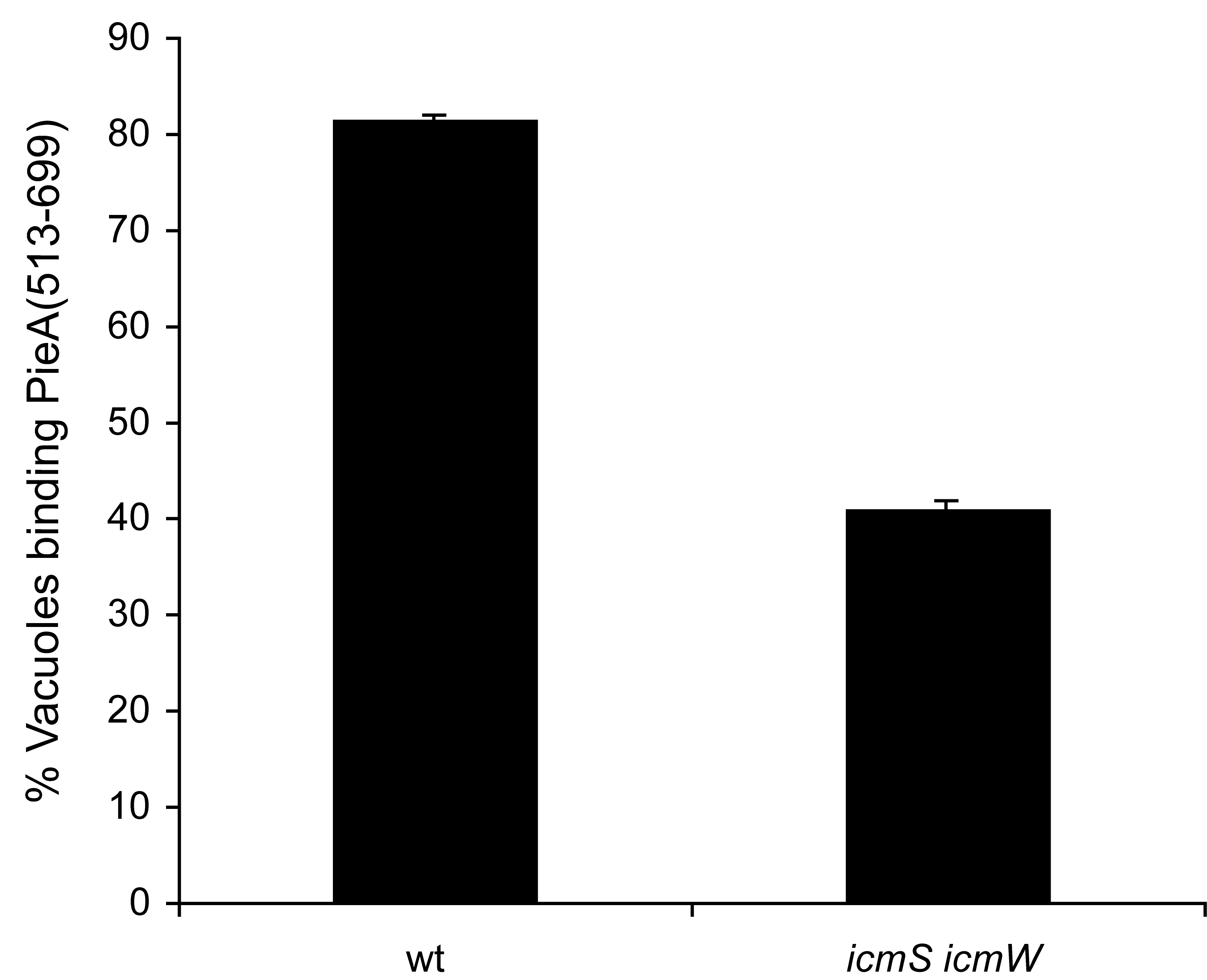

Supplement: Figure S4 — PieA binding to vacuoles containing L. pneumophila is impaired in the absence of the IcmS-IcmW complex. L. pneumophila vacuoles were isolated from U937 macrophage-like cells infected with either wild-type bacteria (wt), or a mutant strain lacking the icmS and icmW genes (icmS icmW). Vacuoles were incubated with purified PieA(513–699) protein and bound protein was detected using a polyclonal antibody directed against PieA. L. pneumophila containing vacuoles were scored to quantify the percent of vacuoles that bound to PieA. Values are means±standard-error of mean for three independent experiments. (7.89 MB TIF) [file ppat.1000278.s005.tif]
